# Effect of sodium 4-phenylbutyrate on Clenbuterol-mediated muscle growth

**DOI:** 10.1371/journal.pone.0201481

**Published:** 2018-07-27

**Authors:** David M. Brown, Sarah Jones, Zoe C. T. R. Daniel, Madelaine C. Brearley, Jo E. Lewis, Francis J. P. Ebling, Tim Parr, John M. Brameld

**Affiliations:** 1 School of Biosciences, University of Nottingham, Sutton Bonington Campus, Loughborough, United Kingdom; 2 School of Life Sciences, University of Nottingham, Medical School, Nottingham, United Kingdom; University of Louisville School of Medicine, UNITED STATES

## Abstract

Previously, we highlighted induction of an integrated stress response (ISR) gene program in skeletal muscle of pigs treated with a beta-adrenergic agonist. Hence we tested the hypothesis that the ER-stress inhibitor, sodium 4-phenylbutyrate (PBA), would inhibit Clenbuterol-mediated muscle growth and reduce expression of genes that are known indicators of an ISR in mice. Clenbuterol (1mg/kg/day) administered to C57BL6/J mice for 21 days increased body weight (*p*<0.001), muscle weights (*p*<0.01), and muscle fibre diameters (*p*<0.05). Co-administration of PBA (100mg/kg/day) did not alter the Clenbuterol-mediated phenotype, nor did PBA alone have any effects compared to that of the vehicle treated mice. Clenbuterol increased skeletal muscle mRNA expression of phosphoserine amino transferase 1 (PSAT1, *p*<0.001) and cyclophillin A (*p*<0.01) at day 3, but not day 7. Clenbuterol decreased mRNA expression of activating transcription factor (ATF) 4 and ATF5 at day 3 (*p*<0.05) and day 7 (*p*<0.01), X-box binding protein 1 (XBP1) variant 2 mRNA at day 3 only (*p*<0.01) and DNA damage inducible transcript 3 (DDIT3/CHOP) mRNA at day 7 only (*p*<0.05). Co-administration of PBA had no effect on Clenbuterol-induced changes in skeletal muscle gene expression. In contrast, treatment of C2C12 myotubes with 5mM PBA (8hr) attenuated the thapsigargin-induced ISR gene program. Prolonged (24-48hr) treatment with PBA caused atrophy (*p*<0.01), reduced neoprotein synthesis (*p*<0.0001) and decreased expression of myogenin and fast myosin heavy chain genes (*p*<0.01), indicating an inhibition of myogenic differentiation. In summary, Clenbuterol did not induce an ISR gene program in mouse muscle. On the contrary, it reduced expression of a number of ISR genes, but it increased expression of PSAT1 mRNA. Co-administration of PBA had no effect on Clenbuterol-mediated muscle growth or gene expression in mice, whereas PBA did inhibit thapsigargin-induced ISR gene expression in cultured C2C12 cells and appeared to inhibit myogenic differentiation, independent of altering ISR gene expression.

## Introduction

Retention of lean body mass is largely achieved by maintaining rates of skeletal muscle protein synthesis and degradation in equilibrium [1]. The ability to retain skeletal muscle mass is impaired with ageing [2], which is associated with reduced mobility and increased all-cause mortality [3–5]. The identification of conserved mechanisms regulating skeletal muscle protein metabolism are required for the development of novel therapeutics controlling muscle mass.

The integrated stress response (ISR) is a highly conserved frontline protection against a diverse array of stressors, such as amino acid limitation, viral infection, or an accumulation of miss-folded proteins [6]. Phosphorylation of eukaryotic translation initiation factor 2 alpha (eIF2α) is considered a central event of the ISR, which decreases global protein synthesis, whilst selectively promoting translation of activating transcription factor 4 (ATF4). The mammalian target of rapamycin (mTORC1), a potent stimulator of protein synthesis [6], can also regulate a post-transcriptional increase in ATF4 expression independent of eIF2α phosphorylation [7]. Depending on the severity of stress, the ISR leads to transcription of functionally related cohorts of ATF4 target genes, which attempt to restore cellular homeostasis or, if homeostasis cannot be resumed, initiate programmed cell death [8]. Genes involved in this adaptive response regulate several aspects of protein metabolism, namely amino acid biosynthesis, protein translation, and amino acid transport. Thus, the ISR is a potentially exploitable mechanism to manipulate skeletal muscle growth and metabolism.

We recently demonstrated induction of an ISR gene program in skeletal muscle of pigs treated with a beta-adrenergic agonist (BA) [9], a potent class of muscle anabolic drug. Specifically, treatment with a BA triggered a co-ordinated and temporal increase in expression of 20+ genes that are known transcriptional targets of ATF4, the best-characterized central effector of the ISR gene program. The majority of these genes were involved in amino acid biosynthesis (*Phgdh*, *Psat1*, *Psph*, *Pck2*, *Asns*, *Arg2*, *Ass1*, *Gpt2*), tRNA charging (*Sars*, *Tars*, *Gars*, *Wars*, *Iars*, *Aars*), and amino acid transport (*Slc3a2*, *Slc7a1*, *Slc5a1*), as well as inhibition of cellular growth (*Sesn2* and *Cdkn1a*). Importantly, expression of these BA-inducible genes peaked 3 days following the initiation of treatment, and declined thereafter. Several other studies have revealed an ISR in skeletal muscle of rodents and humans following exercise [10,11], inactivity [12,13] and hypertrophic growth [14,15]. However, the role and cause of an ISR during skeletal muscle growth remains unknown. It is possible that an increased demand for amino acids (caused by increased protein synthesis) triggers an ISR. Alternatively, an ISR could occur due to an accumulation of miss-folded proteins, i.e. endoplasmic reticulum (ER) stress, caused by over-reaching the protein synthetic capacity of the cell. Although the precise role of the ER in the maintenance of skeletal muscle mass is unknown, ER-stress (induced by treatment with thapsigargin) causes anabolic resistance in cultured muscle cells through blockade of mTOR-mediated protein translation, exemplifying a link between ER homeostasis and protein metabolism in muscle cells [15].

Sodium-4 Phenylbutyrate (PBA) is a short chain aromatic fatty acid, capable of stabilizing miss-folded proteins and therefore limiting ER-stress [16]. Originally discovered as a nitrogen scavenging treatment for urea cycle disorders [17,18], numerous applications of PBA are rapidly emerging [16,19], with pre-clinical and clinical trials examining its therapeutic role in the treatment of spinal muscular atrophy [20], beta-thalassemia [21], cancer [22,23], Huntington’s disease [24], cystic fibrosis [25], type 2 diabetes [26] and immune disorders [27]. The precise mechanisms by which PBA is protective across such a diverse spectrum of pathological states remains poorly understood but the use of PBA to alleviate ER stress constitutes the majority of recently published work on this small molecule, affirming its role as an ER-stress inhibitor in biomedical research [16,19].

Surprisingly, administration of PBA has previously been shown to be detrimental to the maintenance of muscle mass in tumour bearing but also non-tumour bearing mice [28]. This is largely counter-intuitive given that stabilization of protein folding and maintenance of ER-function would be expected to sustain protein homeostasis, not disrupt it, but it does fit with the idea suggested by our observations in BA treated pigs, that a certain degree of ER-stress is required to sustain muscle growth. To explore this further, we tested the hypothesis that Clenbuterol-mediated muscle growth would be inhibited by co-administration of the ER-stress inhibitor, PBA, using a dose (100mg/kg/d) previously shown to inhibit ER-stress in cachexic mice [28]. The identification of conserved mechanisms controlling skeletal muscle mass is required to develop novel therapeutic strategies against muscle wasting conditions, which remains a major unmet clinical need for several pathological conditions [29].

## Materials and methods

### Compounds

For *in vivo* use, Clenbuterol Hydrochloride (CLEN; CAS# 21898-19-1; Sigma Aldrich UK; Lot# BCBQ2163V) and Sodium 4-Phenylbutyrate (PBA; CAS# 1716-12-7; Source BioScience PLC UK; Lot# 31008) were dissolved in Phosphate Buffered Saline (Sigma Aldrich; PBS), filter-sterilized (through a 0.2μm filter) and frozen in aliquots at -20°C. A separate aliquot was used each day. CLEN and PBA were stored as 2x concentrated stocks (0.2μg/μl and 20μg/μl, respectively) and diluted 1:1 with either PBS or the combining compound (i.e. CLEN with PBA) on the day of use.

For *in vitro* use, PBA and Puromycin (Fisher UK) were dissolved in PBS, whereas Thapsigargin (Sigma Aldrich UK) was dissolved in Dimethyl sulfoxide (DMSO). Dissolved compounds were filter-sterilized (through a 0.2μm filter) and frozen in aliquots at -20°C. All compounds were diluted 1:1000 into culture media to achieve 5mM PBA, 1μM Puromcyin, and 250nM Thapsigargin. The corresponding treatment vehicle was used for controls. DMSO accounted for 0.1% (v/v) of the culture media in Thapsigargin experiments.

### Effect of Clenbuterol and PBA *in vivo*

C57BL6/J male mice (approximately 5–6 weeks of age; Charles River UK Ltd.) were singly housed at 21 ± 1°C on a 12hr light: 12hr dark photoperiod (lights off at 19:00), with *ad libitum* access to food and water. Mice were fed Teklad Global 18% Protein Rodent Diet (Envigo). Following 7 days acclimatization to singly housed conditions, mice (*n* = 6 per treatment, per time point) were administered a daily subcutaneous injection (at approximately 9am) for 3, 7 or 21 days with one of the following: Clenbuterol (1mg/kg/day), Clenbuterol (1mg/kg/day) + PBA (100mg/kg/day [28]), PBA only (100mg/kg/day [28]), or the vehicle control (phosphate buffered saline). Body weight and feed intake was monitored daily.

Initial responses to the treatment were carefully monitored for 24 hours in a Comprehensive Lab Animal Monitoring System (CLAMS; Linton Instrumentation, Linton, UK, and Columbus Instruments, Columbus, OH, USA). Changes in physical activity (measured by infrared beam breaks) and feeding behavior permitted deduction of adverse reactions to the administered compound. All animals were acclimatized to the CLAMS system for a 24hr period immediately preceding the 24hr data collection period.

Following 3, 7 or 21 days of treatment, animals were euthanized by cervical dislocation. The *Tibialis Anterior* (TA), *Extensor Digitorum Longus* (EDL), *Gastrocnemius* (GA) and *Soleus* (SOL) muscles were carefully dissected and weighed (wet weight). The GA muscle was snap frozen on solid carbon dioxide, before being stored at -80°C for analysis of gene expression. The TA muscle was coated in optimal cutting temperature (OCT) compound and then snap frozen in dry ice-chilled isopentane, before being stored at -80°C for analysis of muscle fibre diameters.

All animal procedures were approved by The University of Nottingham Ethical Review Committee and conducted in accordance with the UK Animals (Scientific Procedures) Act of 1986 (Project License PPL 40/3604).

### RNA extraction and quantitative-RT-PCR

Using a liquid nitrogen-cooled pestle and mortar, a gastrocnemius muscle from each animal per treatment at day 3 and 7 (*n* = 6 per treatment) was crushed to a fine powder. Total RNA was extracted from 20mg of crushed muscle using the RNeasy Fibrous Tissue Mini Kit (Qiagen) according to manufacturer’s instructions.

Total RNA from cultured C2C12 myotubes was extracted as previously described [30] using the High Pure RNA Isolation Kit (Roche).

First strand cDNA synthesis was performed on 500ng RNA using the Transcriptor First Strand cDNA synthesis Kit (Roche) according to manufacturer’s instructions. Quantitative-RT-PCR was conducted using the SYBR-green method as previously described [30]. Relative transcript abundance was determined from a standard curve and normalised against total cDNA content of the PCR reaction using the established oligreen method [31] as previously described [30]. Oligonucleotide sequences are shown in Table 1. Sequences for myosin heavy chain isoforms and myogenin have been published elsewhere [30].

**Table 1:** Murine oligonucleotide sequences for q-RT-PCR.

Gene	Forward	Reverse
ATF4	TGGCCAAGCACTTGAAACCT	GCCAAGCCATCATCCATAGC
ATF5	GAGAGGGAGGTCTCGTGTACGT	GATGGACTGGATAGGAAAGTGGAA
DDIT1	CCCCGGACCTGCACTGT	GACTTAAGGCAGGATCCTTCCA
DDIT3	GAAGAGGAAGAATCAAAAACCTTCA	GCCCTGGCTCCTCTGTCA
SESN2	TCAGCGAGGTCAACAAGTTACTG	CAAGGCCTGGATATGCTCCTT
CEBPG	GAGGCGCAGGTACATGTGAA	CCTTTGCCAACACAGAATAGGTAGA
PPIA	TGATGGCGAGCCCTTGG	TCTGCTGTCTTTGGAACTTTGTC
PHGDH	CGTGAACTTGGTGAACGCTAAG	GTGGGAGGTGGTGACATTGAG
PSAT1	CGTGCTTCAGCATCTACGTCAT	GCCCCGCCGTTGTTCT
PSPH	GGCATAAGGGAGCTGGTAAGC	GCCACCAGAGATGAGGAACAC
ASNS	TGAAGGAACTCTACCTGTTTGATGTT	GGGACTCTCAGTTCGAGACCGT
PCK2	GCAGAGCACATGCTGATTTTG	GGAAAGCAGCTGCCACGTA
Total XBP1	AAGAACACGCTTGGGAATGG	ACTCCCCTTGGCCTCCAC
XBP1 variant 1 (unspliced)	CCGCAGCACTCAGACTATGTG	CCCAAAAGGATATCAGACTCAGAATC
XBP1 variant 2 (spliced)	GAGTCCGCAGCAGGTG	GTGTCAGAGTCCATGGGA

### Muscle fibre diameter analysis

Sections from the mid-belly of the TA muscle were cryosectioned at -24°C, at a thickness of 12μm. Sections were air-dried for one hour and then stained with 5μg/ml Wheat Germ Agglutinin (WGA) containing an Alexa Fluor® 488 Conjugate (ThermoFisher #W11261) for 20 minutes. Sections were then washed with phosphate buffered saline for 2 x 5 minutes, air-dried and then mounted with Fluoroshield™ containing DAPI overnight. Five fields of view (each containing ~100 fibres) from a single section per muscle were imaged at 20x magnification. Sections from 6 TA muscles per treatment were analysed, which equated to ~3000 fibres per treatment being measured. Muscle fibre perimeters were manually traced and the minimal feret’s diameter was calculated using image-J software. Minimal feret’s diameter of individual muscle fibres is considered superior to measuring muscle fibre cross-sectional area because it is less influenced by differences in the orientation of the muscle when sectioning [32]. The treatment groups were blinded to the investigator during the analysis.

### Cell culture

C2C12 murine skeletal muscle myoblasts [33] were cultured as previously described [30]. Briefly, myoblasts were cultured in growth media (Dulbecco’s Modified Eagle’s Medium (DMEM), 10% fetal bovine serum, 1% penicillin streptomycin; Sigma-Aldrich UK) and encouraged to differentiate at near confluence by switching to differentiation media (DMEM, 2% horse serum, 1% penicillin streptomycin; Sigma-Aldrich UK). Differentiation media was replaced every other day. Cells were incubated at 37°C, with 5% CO_2_ and atmospheric oxygen (~21%). Pharmacological treatments on myotubes were normally conducted on 4-day differentiated cells, whereby substantial cell fusion had already occurred. Treatments added to differentiating myoblasts were initiated at near confluence (day 0) when the media was switched to differentiation medium.

### Myotube size assays

Two methods of myotube size assessment were conducted to robustly characterize the effects of PBA and serum content on myotube atrophy/hypertrophy.

#### Method 1

C2C12 myoblasts were transiently transfected with a *Myh4*-promoter driven ZsGreen reporter construct, as previously described [34]. We have demonstrated that expression of the *Myh4* gene (encoding myosin heavy chain IIB) is restricted to fully differentiated myotubes [30] and activity of the *Myh4* promoter, as detected by a green fluorescent protein (ZsGreen), in living C2C12 cells is a ‘real-time’ biomarker of mature myotubes in culture [34]. Green fluorescing myotubes (independent of nuclei number) were imaged pre-, during and post- treatment to assess transient changes in average myotube width (repeated measures per well). Measurements were conducted (using Image-J) at approximately 25, 50 and 75% of the total myotube length on 10 myotubes per field of view. Five random fields of view per well (on a 24-well plate) were analysed, providing an average myotube width of 50 myotubes per well and 150 myotubes per treatment. The investigator was blinded to the treatment groups during analysis.

#### Method 2

C2C12 myotubes, defined as a MyHC expressing cell containing 2 or more nuclei, were stained, imaged and measured. Briefly, culture media was removed and cells were washed with warm PBS before being fixed in ice-cold methanol for 5 minutes. Cells were washed with room temperature PBS before incubation with primary blocking solution (PBS with 10% horse serum and 0.1% Triton-X; Sigma Aldrich) for 1 hour. Cells were then incubated overnight with the MF20 antibody (1:1000; Developmental Studies Hybridoma Bank, The University of Iowa) against total myosin heavy chain in secondary blocking solution (PBS, 10% horse serum, 0.05% Triton-X). Cells were washed 3 times with PBS for 5 minutes each time, followed by a 2-hour incubation with anti-mouse Alexa Fluor 488 (1:1000; #A21121 Invitrogen) in secondary blocking solution. Cells were washed 3 times with PBS and then incubated with PBS containing propodium iodide (1:3000; Sigma-Aldrich) for 5 minutes to fluorescently label the cell nuclei. Cells were further washed in PBS and then stored in PBS, with protection from light, at 4°C until imaged. Measurements were conducted as described in *Method 1* above. To determine average nuclei number, nuclei were counted in 5 random fields of view per well.

### SUnSET assay on C2C12 cells

Using Puromycin, which incorporates into nascent peptide chains, the SUnSET assay [35] was used herein to quantify global neoprotein synthesis in cultured cells. Following 24 or 48hrs of treatment, puromycin was added to the culture media (final concentration of 1μM) for exactly 30 minutes prior to harvesting the cells in 150μl 1x Laemmli buffer (containing protease and phosphatase inhibitors; Roche) per well of a 24-well plate. Protein concentration was normalised to a constant weight/volume and 10μg protein was electrophoresed on 4–15% acrylamide gels (BioRad Criterion TGX). Proteins were wet-transferred to a nitrocellulose membrane, probed for anti-puromycin (1:1000; MerkMillipore MABE343, clone 12D10) overnight, followed by anti-mouse (1:25000; GE Health Care) for 90 minutes. Bands were visualised using ECL select detection reagent (GE Health Care) and exposing the membrane to photographic film in a dark room. Densitometry of each lane (i.e. all bands) indicated the amount of puromycin incorporated into newly synthesized proteins within a 30-minute window.

### Statistical analyses

Statistical analyses were performed using Prism (Version 7). All data generated by *in vivo* experiments were assessed by a two-way analysis of variance (ANOVA) for significant effects of treatment, time or an interaction (treatment x time; treatment x treatment). Cell culture experiments were assessed by unpaired t-tests (if using a single treatment at a single time point) or by two-way ANOVA (if using multiple time points or combined treatments). Sidak’s or Tukey’s post hoc analysis was conducted where appropriate. Statistical significance was accepted if *p*<0.05. All data are presented as mean ± standard error of the mean.

## Results

### PBA does not modify Clenbuterol-mediated improvements in weight gain and feed efficiency

**Fig 1:**
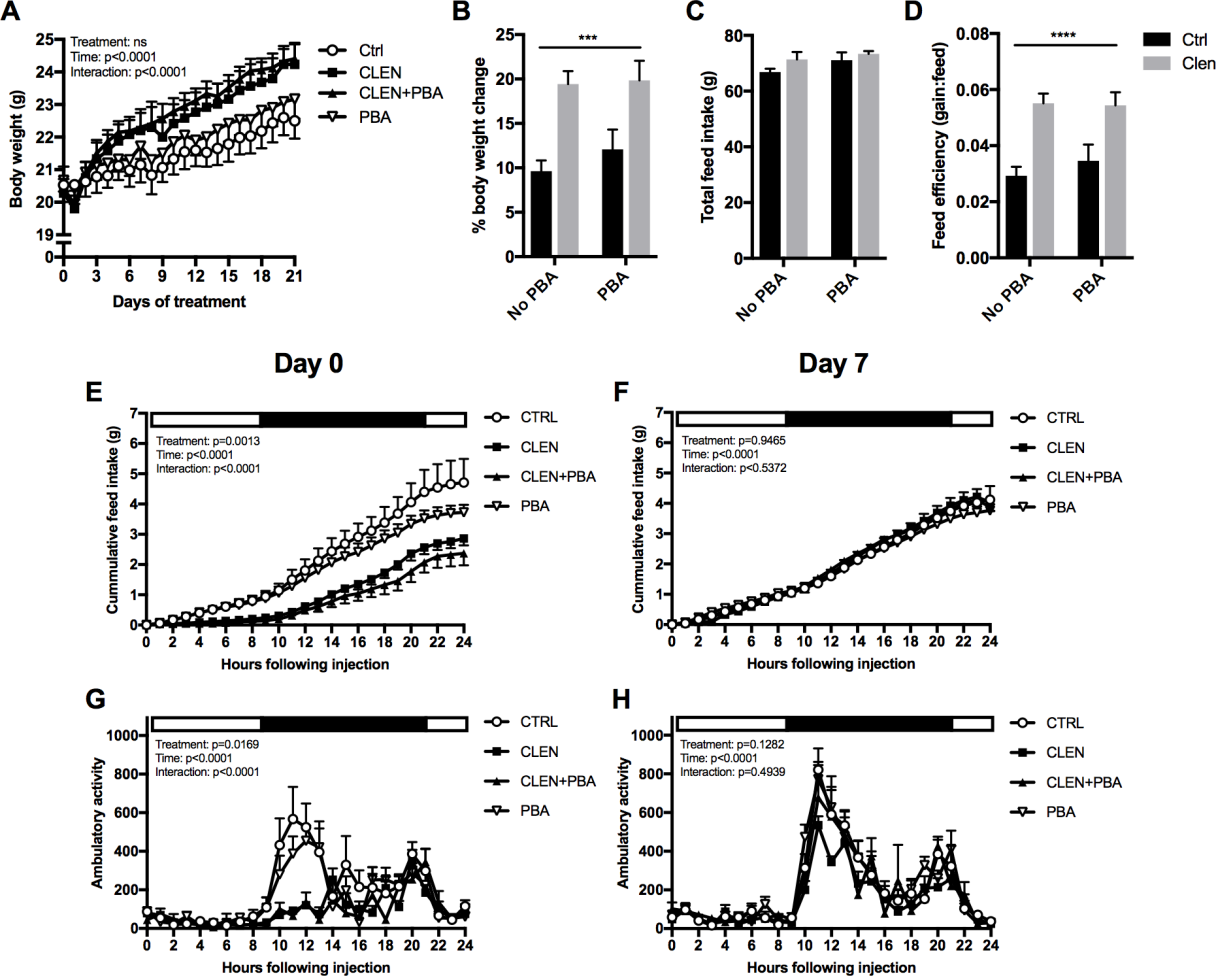
Effect of Clenbuterol and PBA on weight gain, feed efficiency, feeding behaviour and ambulatory activity. (A) Body weight change with treatment and time. (B) Overall percentage body weight change following 21 days of treatment. (C) Total feed consumed during 21 days of treatment. (D) Gross feed efficiency (total body weight change divided by total feed consumed). (E) 24-hour cumulative food intake following initial drug administration (day 0). (F) 24-hour cumulative food intake following drug administration on day 7. (G) 24-hour ambulatory activity following initial drug administration (day 0). (H) 24-hour ambulatory activity following drug administration on day 7. Asterisks denote a significant main effect of Clenbuterol: *** *p*<0.001 **** *p*<0.0001. There was no significant effect of PBA.

Despite no change in food intake, mice administered daily injections of Clenbuterol for 21 days, with or without PBA, gained approximately twice as much weight as the control or PBA-only treated mice (Fig 1A–1C; *p*<0.001). Consequently, gross feed conversion efficiency (gain:feed) was improved approximately 2-fold with Clenbuterol (Fig 1D; *p*<0.0001), with no effect of PBA.

Unexpectedly, the initial administration of Clenbuterol, with or without PBA, caused a temporary reduction in 24-hour food intake (Fig 1E; interaction *p*<0.0001) and ambulatory activity (Fig 1G; interaction *p*<0.0001), which had not previously been described. This response was not apparent when the same parameters were assessed at day 7 (Fig 1F–1H), neither were they apparent from the home cage food intake data on day 2 (data not shown).

### PBA does not modify Clenbuterol-mediated increases in muscle mass

**Fig 2:**
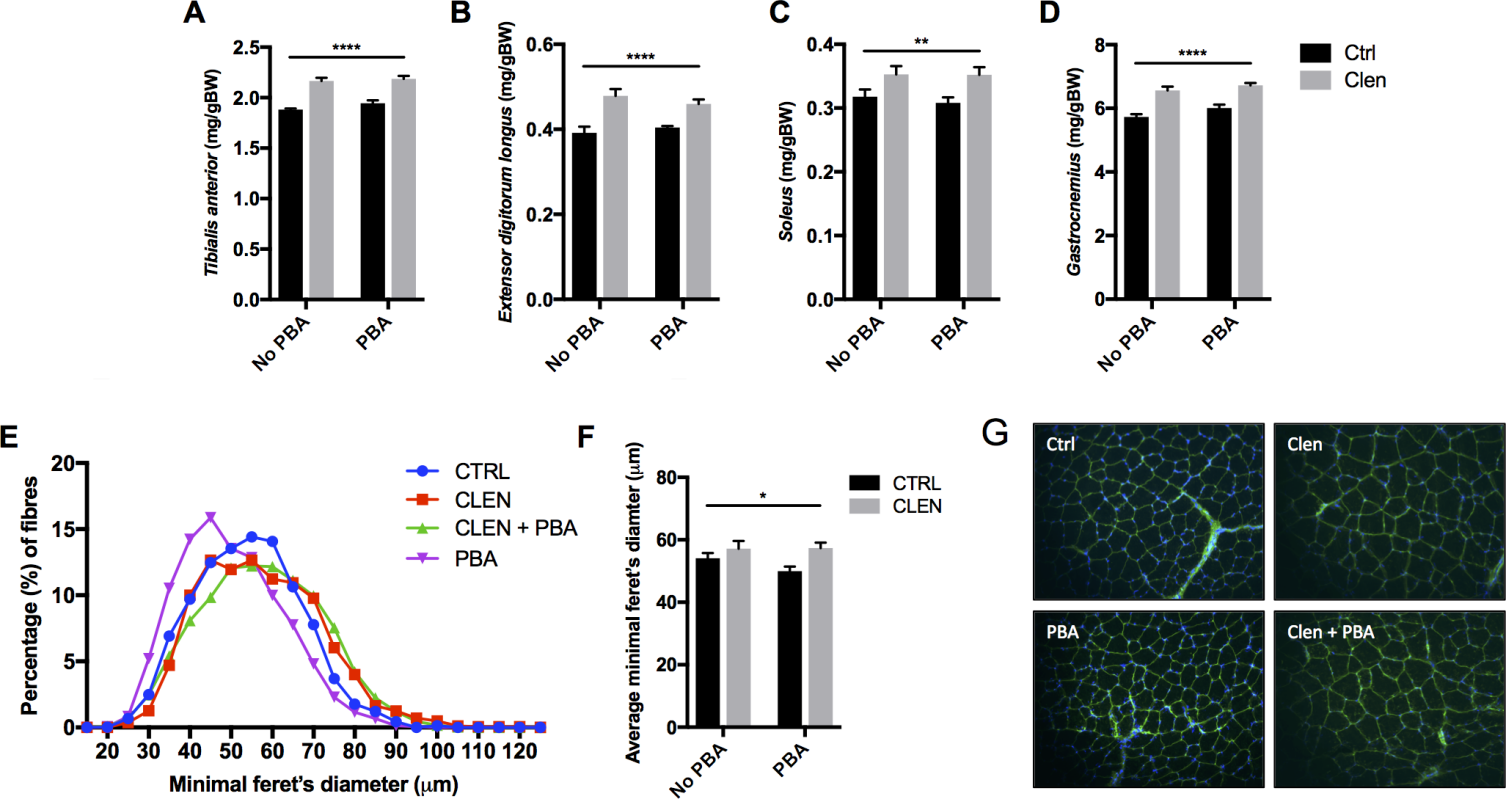
Effect of Clenbuterol and PBA on individual muscle weights and TA muscle fibre diameters. (A) Relative *Tibalis anterior* muscle weight (milligrams per gram of body weight). (B) Relative *Extensor digitorum longus* muscle weight (milligrams per gram of body weight). (C) Relative *Soleus* muscle weight (milligrams per gram of body weight). (D) Relative *Gastrocnemius* muscle weight (milligrams per gram of body weight). (E) Percentage frequency distribution of muscle fibre sizes analysed in *Tibalis anterior* muscle. (F) Average minimal feret’s diameter of *Tibalis anterior* muscle fibres. (G) Representative images of WGA-stained *Tibalis anterior* muscle fibre cross-sections (20x magnification). Asterisks denote a significant main effect of Clenbuterol: * *p*<0.05, ** *p*<0.01, **** *p*<0.0001. There was no significant effect of PBA.

Clenbuterol significantly (*p*<0.01) increased the weight of individual hind limb muscles (*Tibialis anterior*, *Extensor digitorum longus*, *Soleus*, and *Gastrocnemius*), with no effect of co-administering PBA (Fig 2A–2D). This was confirmed by an increase in the average minimal feret’s diameter of *Tibalis anterior* muscle fibres from Clenbuterol treated animals, with no effect of PBA (Fig 2E–2G). Whilst PBA alone did not significantly (*p*>0.05) decrease the average muscle fibre diameter in comparison to that of the control, there was a notable leftward shift in the frequency of smaller muscle fibres at the expense of larger ones compared to that of the control and Clenbuterol treated groups (Fig 2E).

### Molecular effects of Clenbuterol and PBA on expression of integrated stress-responsive (ISR) genes

Previously published data demonstrated that treatment with a beta-adrenergic agonist induced increased expression of amino acid biosynthesis and other integrated stress-responsive (ISR) genes in pig skeletal muscle, peaking between days 3 and 7 of treatment [9]. We therefore assessed changes in expression of these genes at the same time points in mice treated with Clenbuterol (with and without PBA).

**Fig 3:**
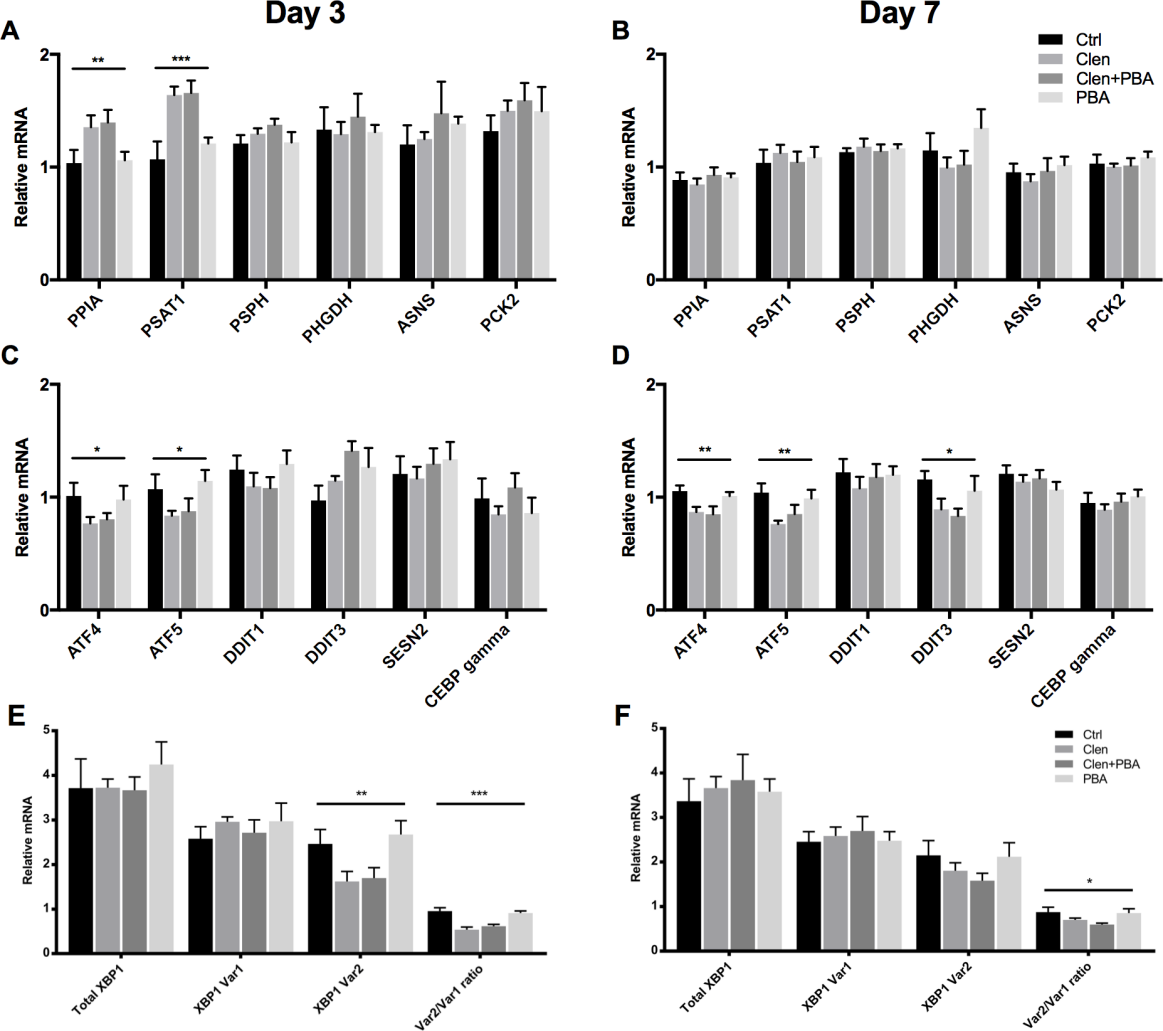
Effect of Clenbuterol and PBA on the expression of metabolic and stress-responsive genes in mouse muscle. (A) Day 3 mRNA abundance of Cyclophillin (CYCLO), Phosphoserine aminotransferase 1 (PSAT1), Phosphoserine phosphatase (PSPH), 3-Phosphoglycerate dehydrogenase (PHGDH), asparagene synthetase (ASNS), mitochondrial phosphoenolpyruvate carboxykinase (PCK2). (B) Day 7 mRNA abundance of genes listed above (see A). (C) Day 3 mRNA abundance of Activating transcription factor 4 (ATF4), Activating transcription factor 5 (ATF), Growth arrest and DNA damage inducible factor 45 alpha (DDIT1), Growth arrest and DNA damage inducible protein 153 (DDIT3), Sestrin-2 (SESN2). (D) Day 7 mRNA abundance of genes listed above (see C). (E) Day 3 mRNA abundance of Total X-box binding protein 1 (XBP1), unspliced Variant 1 XBP1 (XBP1 Var1), spliced variant 2 XBP1 (XBP1 Var2) and the Variant ratio (Var 2/Var 1). (F) Day 7 mRNA abundance of genes listed above (see E). Asterisks denote a significant main effect of Clenbuterol: * *p*<0.05, ** *p*<0.01, *** *p*<0.001, **** *p*<0.0001. There was no significant effect of PBA.

Of the serine biosynthesis pathway genes (*Phgdh*, *Psat1* and *Psph*), only *Psat1* displayed a significant increase in mRNA expression by Clenbuterol at day 3 (*p*<0.001), but not day 7, with no effect of co-administering PBA (Fig 3A and 3B). Cyclophilin A (*Ppia*), which is involved in protein folding, was also increased by Clenbuterol treatment at day 3 (*p*<0.01), but not day 7, with no effect of PBA (Fig 3A and 3B). Other metabolic genes involved in amino acid biosynthesis and gluconeogenesis, such as *Asns* and *Pck2*, displayed no change in expression in response to Clenbuterol or PBA treatment (*p*>0.05; Fig 3A and 3B).

In contrast to our expectations based on previous work in pigs [9], Clenbuterol significantly decreased mRNA expression of the stress-responsive transcription factors *Atf4* and *Atf5* at days 3 (*p*<0.05) and 7 (*p*<0.01), with no effect of PBA (Fig 3C and 3D). Gene expression of another stress-responsive transcription factor, *Ddit3* (also known as *Chop*), was decreased by Clenbuterol treatment at day 7 (*p*<0.05) but not day 3, with no effect of PBA (Fig 3C and 3D). Other stress-responsive genes, *Ddit1*, *Sesn2* and *Cebpg*, did not change with PBA or Clenbuterol treatment (*p*>0.05; Fig 3C and 3D), nor did PBA or Clenbuterol alter the expression of X-box binding protein 1 (XBP1) variant 1 or Total XBP1 mRNA (Fig 3E and 3F), although Clenbuterol did significantly reduce XBP1 variant 2 mRNA on day 3 (*p* = 0.004, Fig 3E), the spliced variant induced as part of an ER stress response, as well as the ratio of variant 2: variant 1 on days 3 (*p*<0.001; Fig 3E) and 7 (*p* = 0.011; Fig 3F).

### PBA inhibits the expression of integrated stress-responsive (ISR) genes in cultured myotubes

**Fig 4:**
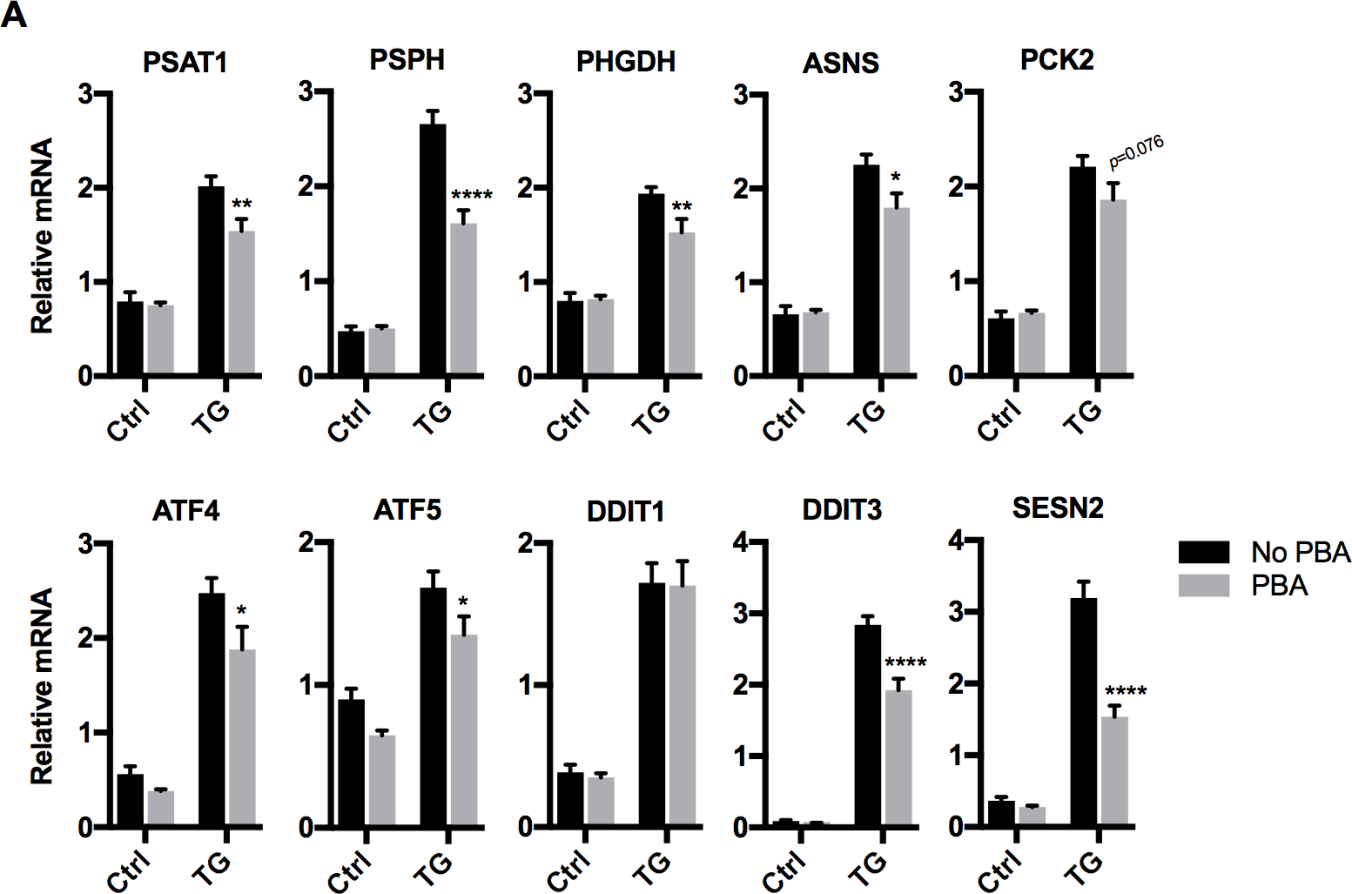
Effect of 5mM PBA on the expression of thapsigargin-inducible (ER-stress) genes in C2C12 myotubes. (A) mRNA abundance of Phosphoserine aminotransferase 1 (PSAT1), Phosphoserine phosphatase (PSPH), 3-Phosphoglycerate dehydrogenase (PHGDH), asparagine synthetase (ASNS), mitochondrial phosphoenolpyruvate carboxykinase (PCK2), Activating transcription factor 4 (ATF4), Activating transcription factor 5 (ATF), Growth arrest and DNA damage inducible factor 45 alpha (DDIT1), Growth arrest and DNA damage inducible protein 153 (DDIT3), Sestrin-2 (SESN2). C2C12 myotubes (4-day differentiated) were treated with 250nM Thapsigargin (TG) in the presence or absence of 5mM sodium 4-phenylbutyrate (PBA) for 8 hours. Expression of all genes was significantly increased by thapsigargin (TG) treatment (*p*<0.0001). With the exception of DDIT1 and PCK2, there was a significant effect of PBA on TG-induced expression of all genes (*p*<0.05). Asterisks denote the post-hoc Sidak’s multiple comparisons test for a significant effect of PBA relative to No PBA within the control or Thapsigargin treated groups: * *p*<0.05, ** *p*<0.01, **** *p*<0.0001.

In order to confirm that the PBA used was capable of inhibiting ER stress-induced gene expression, we determined its effects on thapsigargin-induced ER stress *in vitro*. C2C12 myotubes were treated for 8 hours with 250nM thapsigargin, which caused a significant (*p*<0.0001) increase in mRNA expression of several ISR genes (*Psat1*, *Psph*, *Phgdh*, *Asns*, *Pck2*, *Atf4*, *Atf5*, *Ddit1*, *Ddit3*, *Sesn2*). As expected, co-treatment with 5mM PBA significantly (*p*<0.05) attenuated, but did not completely block, the thapsigargin-induced increase in mRNA expression of the majority of ISR genes (Fig 4A), with the exception of *Ddit1*. In contrast, the addition of PBA alone, in the absence of thapsigargin, had no effect on the ISR gene expression (*p*>0.05; Fig 4A).

### Effects of PBA on myotube size and myogenic differentiation in C2C12 cells

**Fig 5:**
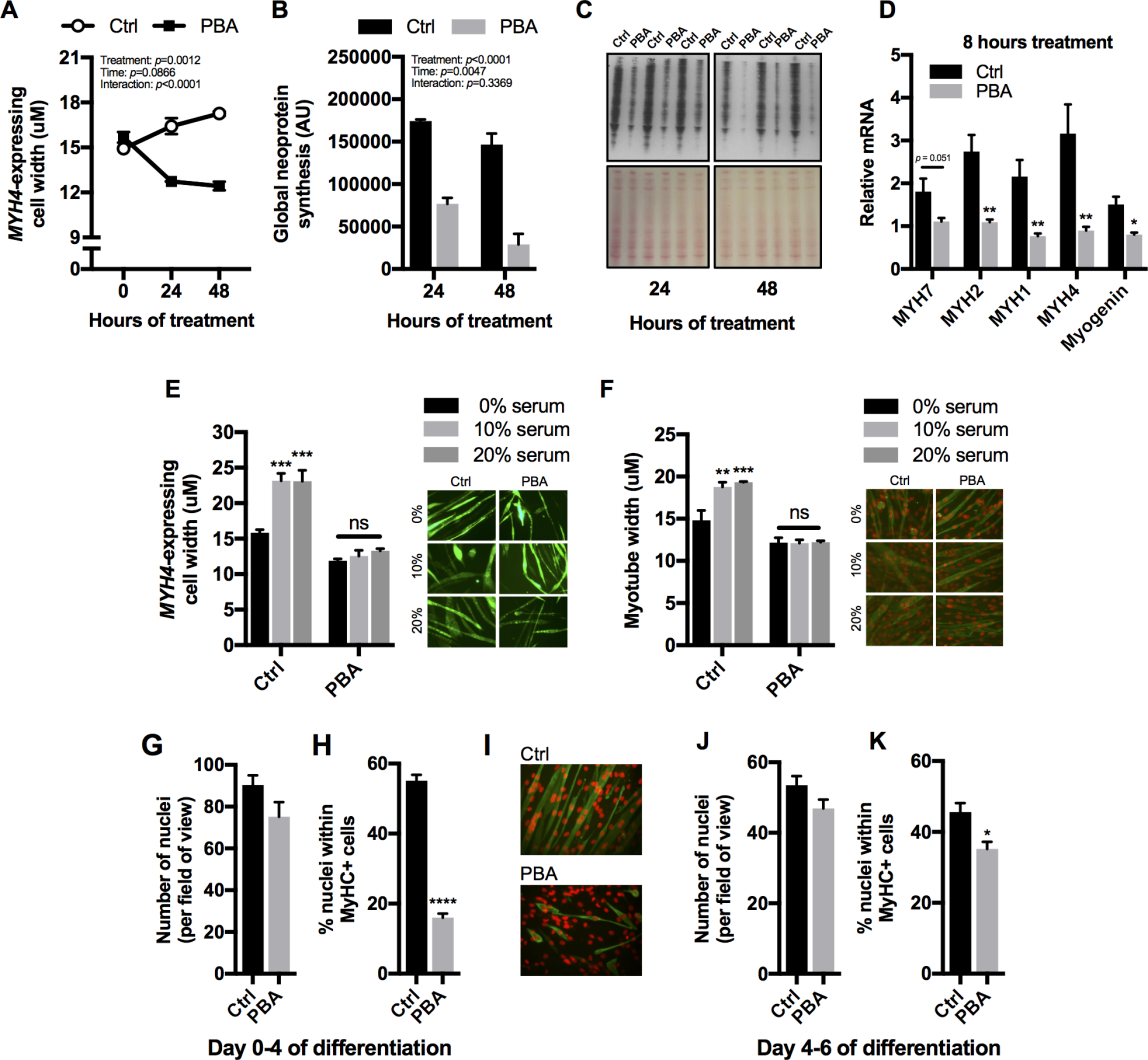
Effects of PBA on C2C12 myotube size and differentiation. (A) Myotube width of MYH4 expressing cells following 0, 24 and 48 hours of treatment with 5mM sodium 4-phenylbutuyrate (PBA). Measurements conducted on live cells expressing ZsGreen transcribed from an MYH4 promoter. (B) Global neoprotein synthesis (assessed by the relative incorporation of puromycin into nascent proteins) following 24 and 48 hours of treatment with 5mM PBA. (C) Representative images of puromycin incorporation (for quantification see B). (D) Relative expression of myosin heavy chain isoforms (MYH7, MYH2, MYH1, MYH4) and myogenin mRNA in myotubes treated with 5mM PBA for 8 hours. (E) Effect of differential fetal serum content (0, 10 and 20%) in the presence or absence of 5mM PBA on myotube width. Measurements were conducted on live cells expressing ZsGreen transcribed from an MYH4 promoter. (F) Effect of differential serum content (0, 10 and 20%) in the presence and absence of 5mM PBA on myotube width. Measurements were conducted on fixed cells stained for myosin heavy chain (using MF20—green) and nuclei (using propodium iodide—red). (G) Number of nuclei per field of view and (H) Percentage of nuclei residing in myosin heavy chain positive cells (myotubes) following treatment with Vehicle (Ctrl) or 5mM PBA between days 0 and 4 of differentiation. (I) Representative images of Vehicle (Ctrl) and PBA (5mM) treated C2C12 cells at day 4 of differentiation following staining for myosin heavy chain (using MF20—green) and nuclei (using propodium iodide—red). (J) Number of nuclei per field of view and (K) Percentage of nuclei residing in myosin heavy chain positive cells (myotubes) following treatment with Vehicle (Ctrl) or 5mM PBA between days 4 and 6 of differentiation. Asterisks in (D), (H) and (K) indicate a significant difference to the Ctrl (T-test), whereas asterisks in (E) and (F) indicate a significant difference to 0% serum within the same treatment group (2-way ANOVA followed by post-hoc Tukey’s multiple comparisons test): * *p*<0.05, ** *p*<0.01, *** *p*<0.001.

Although treatment with PBA *in vivo* had no significant effect on muscle weight or fibre size, the addition of PBA (5mM) to the culture media of terminally differentiated C2C12 myotubes for 24 or 48 hours (repeated measures) resulted in significant myotube atrophy (*p*<0.0012), whereas the vehicle treated myotubes maintained their size during this time frame (Fig 5A). Global neoprotein synthesis was significantly decreased by PBA treatment at 24 and 48 hours (Fig 5B and 5C; *p*<0.0001). An 8-hour treatment of differentiated myotubes with PBA revealed a rapid and significant decrease in mRNA expression of all adult myosin heavy chain (MyHC) isoforms (*Myh7*, *Myh2*, *Myh1*, *Myh4*) and also myogenin (Fig 5D), indicating that PBA might impair the myogenic differentiation of C2C12 cells. Following treatment with PBA from day 0 or day 4 of differentiation, there were no significant (*p*>0.05) differences in nuclei number (Fig 5G and 5H), but there was a significant (*p*<0.05) reduction in the number of nuclei residing within myosin heavy chain positive cells (Fig 5H and 5K), particularly when added from day 0 (Fig 5H and 5I), again suggesting that PBA inhibits myogenic differentiation.

We also assessed whether the addition of fetal bovine serum (which is rich in growth factors) could alleviate PBA-induced myotube atrophy. Irrespective of serum content (0, 10 or 20% fetal serum), PBA treated myotubes were similar in size (*p*>0.05), whereas vehicle treated myotubes were larger in the presence of 10 or 20% serum compared to 0% (Fig 5E and 5F; *p*<0.01). This was confirmed using the 2 different methods of myotube size quantification, the *Myh4*-promoter reporter system (Fig 5E) and MyHC/nuclei staining (Fig 5F).

## Discussion

Our previous work highlighted induction of an integrated stress response (ISR) gene program in skeletal muscle of pigs treated with a beta-adrenergic agonist [9]. An ISR in the context of muscle growth is indicative of a perturbation to cellular homeostasis, likely caused by amino acid limitation or endoplasmic reticulum (ER) stress. Hence we tested the hypothesis that the ER-stress inhibitor, sodium 4-phenylbutyrate (PBA), would inhibit Clenbuterol-mediated muscle growth in mice.

Firstly, co-administration of PBA to mice had no effect on the Clenbuterol-mediated increase in muscle mass, muscle fibre diameter or feed conversion efficiency. Secondly, and unexpectedly, with the exception of an increase in *Psat1* mRNA expression, Clenbuterol treatment (for 3 or 7 days) in mice failed to increase any of the 20+ ISR genes that were increased by beta-adrenergic agonist treatment in pigs at the same time points [9]. On the contrary, expression of several ISR genes, including *Atf4*, *Atf5*, *XBP1 variant 2* and *Ddit3*, were actually significantly decreased by Clenbuterol treatment in mice. These data imply that, unlike pigs, mice do not exhibit an ISR in response to administration of a beta-adrenergic agonist. This highlights that translation of molecular events in murine skeletal muscle might not be representative of that in large mammals. Indeed, others have shown that the skeletal muscle transcriptome of mice administered with Clenbuterol (at days 1 and 10, time points different to those used herein) [36] differed to the responses seen in pigs treated with a similar beta-adrenergic receptor agonist (Ractopamine) [37]. Whilst it is clear that PBA does not influence Clenbuterol mediated muscle growth in mice, the lack of a noticeable ISR gene program induced by Clenbuterol makes it difficult to conclude that PBA would not influence Clenbuterol-mediated muscle growth in a large animal species, such as pigs, where a substantial ISR gene program was observed [9,37].

There are several potential reasons for the disparity in ISR gene program induction in studies using rodents versus pigs, including the fact that different beta-adrenergic agonists have been employed (Clenbuterol versus Ractopamine) and that the route of drug administration was different (injected versus oral). However, we suspect the disparity is more likely due to differences in the metabolism of mice compared to large mammals [38,39]. For instance, growing mice and pigs have an ~3% and ~40% feed conversion efficiency, respectively, illustrating a vast difference in basal metabolic rate between small and large mammals. Whilst increasing the rate of growth (which was increased approximately 2-fold by Clenbuterol) is a metabolically demanding/stressful event, it is possible that small mammals, such as mice, are metabolically equipped to deal with such changes more readily than large mammals (like pigs or humans). Indeed, unpublished data from our laboratory suggest that basal expression of several ISR genes (particularly serine biosynthesis pathway genes) are more highly expressed in mice versus pigs. As such, we suggest that mice might therefore not require the adaptive molecular responses to beta-adrenergic agonists that have previously been observed in large mammals, such as pigs [9,37].

Similar doses of PBA to those used herein (100mg/kg/day), ranging from 10mg/kg/day [40] to 100mg/kg/day [41–43], have all previously been shown to attenuate an ISR *in vivo*, but only when an ER stress was actually induced. Furthermore, we showed that PBA could limit induction of an ISR gene program in cultured muscle cells, but only when a known ER-stress inducer (thapsigargin) was present. As such, we are confident that if an ISR had occurred during Clenbuterol-mediated muscle growth, the dose of PBA used was sufficient to attenuate such a response. It is noteworthy however, that previously published literature [40,41,44] indicates that PBA does not suppress expression of ISR genes in the absence of induced-stress, both *in vivo* and *in vitro*, which is presumably why we saw no effects of the PBA *in vivo*.

When testing the efficacy of PBA to block a thapsigargin-induced ISR gene program *in vitro*, we noticed that prolonged treatment with PBA alone caused profound myotube atrophy, which is in agreement with a recent *in vivo* study [28]. Whilst 21 days of PBA treatment *in vivo* did not cause statistically significant muscle atrophy, there was a noticeable reduction in muscle fibre diameters with PBA compared to that of the controls and other treatments. Bohnert et al [28] showed that when the same dose of PBA was administered daily to mice for 28 days, it significantly decreased muscle mass and muscle fibre diameters. It is possible that 3 weeks of PBA treatment is insufficient to observe a significant muscle atrophy phenotype *in vivo*, but that longer (28d) administration of PBA is indeed detrimental to the maintenance of muscle mass. This has serious implications for patients receiving chronic doses of PBA for the treatment of urea cycle disorders, suggesting that they should be monitored accordingly.

When examined *in vitro*, we, and others [28], show that PBA inhibits global neoprotein synthesis in muscle cells, which is most likely responsible for the reduction in myotube size. Expanding on this, we show that PBA drastically inhibits mRNA expression of myogenin and all myosin heavy chain isoforms *in vitro* within 8 hours of treatment, during which, expression of ISR genes was not altered. This implies that PBA affects myogenic differentiation in cultured muscle cells, independent of altering an ISR gene program. It is noteworthy however, that whilst a high dose of PBA (5mM) is required to attenuate ER-stress in cultured cells [28,40,44,45], *in vivo* concentrations of 5mM PBA in the muscle are unlikely. The plasma half-life of PBA in mice is very short (within minutes for moderate doses) [46]. Even when high doses of PBA (36g/day) were administered to humans, plasma PBA concentrations only reached ~1mM, which lasted less than 4 hours [23]. However, *in vitro* data generated by us, and others [28,40,44,45], using high (5mM) PBA concentrations may be representative of chronic exposure to PBA *in vivo*. Another key difference that might relate to differential responses to PBA is that *in vivo* skeletal muscle consists mainly of terminally differentiated muscle fibres, whereas *in vitro* cultures of C2C12 cells are a mix of early stage differentiated myotubes and undifferentiated myoblasts. Since PBA was shown to inhibit myogenic differentiation *in vitro*, then similar effects are unlikely in adult animals, where myogenic differentiation is virtually complete. However, there may be effects observed if PBA were administered *in vivo* during fetal development or after muscle damage when satellite cells are activated and undergo myogenic differentiation.

Finally, we show that the initial administration of Clenbuterol to mice, with or without PBA, drastically inhibited food intake and delayed the onset of ambulatory activity in the dark phase. To the best of our knowledge, this very early response to Clenbuterol has not previously been reported. Accordingly, any metabolic or molecular analyses conducted on mice within 24 hours of initial administration of this drug should be interpreted with caution, as they are likely to be influenced by these behavioural and consequent metabolic changes.

In summary, contrary to our hypothesis Clenbuterol did not induce an integrated stress response (ISR) gene program in mouse muscle, but it did increase PSAT1 mRNA expression, as seen in our previous pig study. Indeed, Clenbuterol actually reduced expression of several ISR genes in mouse skeletal muscle. Co-administration of PBA had no effect on Clenbuterol-mediated muscle growth or gene expression in mice, but PBA did inhibit the thapsigargin-induced ISR gene expression in cultured C2C12 muscle cells and appeared to inhibit myogenic differentiation independent of altering ISR gene expression. There are clear differences between mice and pigs in the molecular responses to beta-agonists, as well as differences between C2C12 muscle cells and mouse skeletal muscle in their response to PBA.

## References

[1] Schiaffino S, Dyar K, Ciciliot S, Blaauw B, Sandri M. Mechanisms regulating skeletal muscle growth and atrophy. FEBS J. 2013;280: 4294–314. 10.1111/febs.12253 23517348

[2] Brown DM, Goljanek-whysall K. microRNAs: Modulators of the underlying pathophysiology of sarcopenia? Ageing Res Rev. Elsevier B.V.; 2015;24: 263–273. 10.1016/j.arr.2015.08.007 26342566

[3] Visser M, Goodpaster BH, Kritchevsky SB, Newman AB, Nevitt M, Rubin SM, et al Muscle mass, muscle strength, and muscle fat infiltration as predictors of incident mobility limitations in well-functioning older persons. J Gerontol A Biol Sci Med Sci. 2005;60: 324–333. 10.1093/gerona/60.3.324 15860469

[4] Landi F, Cruz-Jentoft AJ, Liperoti R, Russo A, Giovannini S, Tosato M, et al Sarcopenia and mortality risk in frail olderpersons aged 80 years and older: Results from iLSIRENTE study. Age Ageing. 2013;42: 203–209. 10.1093/ageing/afs194 23321202

[5] Srikanthan P, Karlamangla AS. Muscle Mass Index As a Predictor of Longevity in Older Adults. Am J Med. Elsevier Inc; 2014;127: 547–553. 10.1016/j.amjmed.2014.02.007 24561114 PMC4035379

[6] Laplante M, Sabatini DM. mTOR signaling in growth control and disease. Cell. Elsevier; 2013;149: 274–293. 10.1016/j.cell.2012.03.017.mTOR PMC3331679 22500797

[7] Park Y, Reyna-Neyra A, Philippe L, Thoreen CC. mTORC1 Balances Cellular Amino Acid Supply with Demand for Protein Synthesis through Post-transcriptional Control of ATF4. Cell Rep. 2017;19: 1083–1090. 10.1016/j.celrep.2017.04.042 28494858 PMC5811220

[8] Pakos-Zebrucka K, Koryga I, Mnich K, Ljujic M, Samali A, Gorman AM. The integrated stress response. EMBO Rep. 2016;17: 1374–1395. 10.15252/embr.201642195 27629041 PMC5048378

[9] Brown DM, Ryan KJ, Daniel ZC, Mareko MH, Talbot R, Moreton J, et al Abstract: 5–03 (Identification of an ER-stress-like response to the anabolic agent, Ractopamine) of the 9th International Conference on Cachexia, Sarcopenia and Muscle Wasting, Berlin, Germany, 10–11 December 2016 (part 1). J Cachexia Sarcopenia Muscle. 2016;7: 626–662. 10.1002/jcsm.12164

[10] Kim HJ, Jamart C, Delidicque L, An G-L, Lee YH, Kim CK, et al Endoplasmic Reticulum Stress Markers and Ubiquitin-Proteasome Pathway Activity in Response to a 200-km Run. Med Sci Sport Exerc. 2011;43: 18–25. 10.1249/MSS.0b013e3181e4c5d1 20473228

[11] Wu J, Ruas JL, Estall JL, Rasbach KA, Choi JH, Ye L, et al The unfolded protein response mediates adaptation to exercise in skeletal muscle through a PGC-1α /ATF6α complex. Cell Metab. 2012;13: 160–169. 10.1016/j.cmet.2011.01.003.The PMC3057411 21284983

[12] Alibegovic AC, Sonne MP, Hojbjerre L, Bork-Jensen J, Jacobsen S, Nilsson E, et al Insulin resistance induced by physical inactivity is associated with multiple transcriptional changes in skeletal muscle in young men. AJP Endocrinol Metab. 2010;299: E752–E763. 10.1152/ajpendo.00590.2009 20739510

[13] Fox DK, Ebert SM, Bongers KS, Dyle MC, Bullard SA, Dierdorff JM, et al p53 and ATF4 mediate distinct and additive pathways to skeletal muscle atrophy during limb immobilization. AJP Endocrinol Metab. 2014;307: E245–E261. 10.1152/ajpendo.00010.2014 24895282 PMC4121573

[14] Hamilton DL, Philp A, MacKenzie MG, Patton A, Towler MC, Gallagher IJ, et al Molecular brakes regulating mTORC1 activation in skeletal muscle following synergist ablation. Am J Physiol Endocrinol Metab. 2014;307: E365–73. 10.1152/ajpendo.00674.2013 24961241 PMC4137116

[15] Deldicque L, Bertrand L, Patton A, Francaux M, Baar K. ER stress induces anabolic resistance in muscle cells through PKB-induced blockade of mTORC1. PLoS One. 2011;6: e20993 10.1371/journal.pone.0020993 21698202 PMC3116857

[16] Kolb PS, Ayaub EA, Zhou W, Yum V, Dickhout JG, Ask K. The therapeutic effects of 4-phenylbutyric acid in maintaining proteostasis. Int J Biochem Cell Biol. 2015;61: 45–52. 10.1016/j.biocel.2015.01.015 25660369

[17] Brusilow S, Danney M, Waber L. Treatment of episodic hyperammonemia in children with inborn errors of urea synthesis. N Engl J Med. 1984;310: 1630–1634. 10.1056/NEJM198406213102503 6427608

[18] Brusilow SW. Phenylacetylglutamine May Replace Urea as a Vehicle for Waste Nitrogen Excretion. Pediatr Res. Nature Publishing Group; 1991;29: 147–150. 10.1203/00006450-199102000-00009 2014149

[19] Iannitti T, Palmieri B. Clinical and Experimental Applications of Sodium Phenylbutyrate. Drugs R D. 2011;11: 227–249. 10.2165/11591280-000000000-00000 21902286 PMC3586072

[20] Mercuri E, Bertini E, Messina S, Pelliccioni M, D’Amico A, Colitto F, et al Pilot trial of phenylbutyrate in spinal muscular atrophy. Neuromuscul Disord. 2004;14: 130–135. 10.1016/j.nmd.2003.11.006 14733959

[21] Collins A, Pearson H, Giardina P, McDonagh K, Brusilow S, Dover G. Oral sodium phenylbutyrate therapy in homozygous beta thalassemia: a clinical trial. Blood. 1995;85. 7528572

[22] Camacho LH, Olson J, Tong WP, Young CW, Spriggs DR, Malkin MG. Phase I dose escalation clinical trial of phenylbutyrate sodium administered twice daily to patients with advanced solid tumors. Invest New Drugs. Kluwer Academic Publishers-Plenum Publishers; 2006;25: 131–138. 10.1007/s10637-006-9017-4 17053987

[23] Gilbert J, Baker SD, Katherine Bowling M, Grochow L, Douglas Figg W, Zabelina Y, et al A Phase I Dose Escalation and Bioavailability Study of Oral Sodium Phenylbutyrate in Patients with Refractory Solid Tumor Malignancies 1. Clin Cancer Res. 2001;7: 2292–2300. 11489804

[24] Gardian G, Browne SE, Choi D-K, Klivenyi P, Gregorio J, Kubilus JK, et al Neuroprotective Effects of Phenylbutyrate in the N171-82Q Transgenic Mouse Model of Huntington’s Disease. J Biol Chem. in Press; 2004;280: 556–563. 10.1074/jbc.M410210200 15494404

[25] Rubenstein R, Zeitlin P. A Pilot Clinical Trial of Oral Sodium 4-Phenylbutyrate (Buphenyl) in Δ F508-Homozygous Cystic Fibrosis Patients: Partial Restoration of Nasal Epithelial CFTR function. Am J Respir Crit Care Med. 1998;157: 4840490. 10.1164/ajrccm.157.2.9706088 9476862

[26] Ozcan U, Yilmaz E, Ozcan L, Furuhashi M, Vaillancourt E, Smith RO, et al Chemical Chaperones Reduce ER Stress and Restore Glucose Homeostasis in a Mouse Model of Type 2 Diabetes. Science (80-). 2006;313: 1137–1140. 10.1126/science.1128294 16931765 PMC4741373

[27] Jellbauer S, Perez Lopez A, Behnsen J, Gao N, Nguyen T, Murphy C, et al Beneficial Effects of Sodium Phenylbutyrate Administration during Infection with Salmonella enterica Serovar Typhimurium. McCormick BA, editor. Infect Immun. 2016;84: 2639–2652. 10.1128/IAI.00132-16 27382022 PMC4995890

[28] Bohnert KR, Gallot YS, Sato S, Xiong G, Hindi SM, Kumar A. Inhibition of ER stress and unfolding protein response pathways causes skeletal muscle wasting during cancer cachexia. FASEB J. 2016; 10.1096/fj.201600250RR 27206451 PMC5001510

[29] von Haehling S, Anker SD. Cachexia as a major underestimated and unmet medical need: facts and numbers. J Cachexia Sarcopenia Muscle. 2010;1: 1–5. 10.1007/s13539-010-0002-6 21475699 PMC3060651

[30] Brown DM, Parr T, Brameld JM. Myosin heavy chain mRNA isoforms are expressed in two distinct cohorts during C2C12 myogenesis. J Muscle Res Cell Motil. 2012;32: 383–90. 10.1007/s10974-011-9267-4 22012579

[31] Rhinn H, Scherman D, Escriou V. One-step quantification of single-stranded DNA in the presence of RNA using Oligreen in a real-time polymerase chain reaction thermocycler. Anal Biochem. 2008;372: 116–118. 10.1016/j.ab.2007.08.023 17963709

[32] Briguet A, Courdier-Fruh I, Foster M, Meier T, Magyar JP. Histological parameters for the quantitative assessment of muscular dystrophy in the mdx-mouse. Neuromuscul Disord. 2004;14: 675–682. 10.1016/j.nmd.2004.06.008 15351425

[33] Yaffe D, Saxel O. Serial passaging and differentiation of myogenic cells isolated from dystrophic mouse muscle. Nature. 270: 725–7. 563524 10.1038/270725a0

[34] Brown DM, Brameld JM, Parr T. Expression of the Myosin Heavy Chain IIB Gene in Porcine Skeletal Muscle: The Role of the CArG-Box Promoter Response Element. PLoS One. 2014;9: 1–17. 10.1371/journal.pone.0114365 25469802 PMC4255089

[35] Schmidt EK, Clavarino G, Ceppi M, Pierre P. SUnSET, a nonradioactive method to monitor protein synthesis. Nat Methods. 2009;6: 275–277. 10.1038/nmeth.1314 19305406

[36] Spurlock DM, McDaneld TG, McIntyre LM. Changes in skeletal muscle gene expression following clenbuterol administration. BMC Genomics. 2006;7: 320 10.1186/1471-2164-7-320 17181869 PMC1766935

[37] Brown DM, Williams H, Ryan KJP, Wilson TL, Daniel ZCTR. Mitochondrial phosphoenolpyruvate carboxykinase (PEPCK-M) and serine biosynthetic pathway genes are co-ordinately increased during anabolic agent-induced skeletal muscle growth. Sci Rep. Nature Publishing Group; 2016; 1–14. 10.1038/s41598-016-0001-8 27350173 PMC4923900

[38] Rennie MJ, Selby a, Atherton P, Smith K, Kumar V, Glover EL, et al Facts, noise and wishful thinking: muscle protein turnover in aging and human disuse atrophy. Scand J Med Sci Sports. 2010;20: 5–9. 10.1111/j.1600-0838.2009.00967.x 19558380

[39] Demetrius L. Of mice and men. When it comes to studying ageing and the means to slow it down, mice are not just small humans. EMBO Rep. 2005;6 Spec No: S39-44. 10.1038/sj.embor.7400422 15995660 PMC1369270

[40] Zeng M, Sang W, Chen S, Chen R, Zhang H, Xue F, et al 4-PBA inhibits LPS-induced inflammation through regulating ER stress and autophagy in acute lung injury models. Toxicol Lett. 2017; 10.1016/j.toxlet.2017.02.023 28245985

[41] Ma J, Luo T, Zeng Z, Fu H, Asano Y, Liao Y, et al Histone Deacetylase Inhibitor Phenylbutyrate Exaggerates Heart Failure in Pressure Overloaded Mice independently of HDAC inhibition. Sci Rep. Nature Publishing Group; 2016;6: 34036 10.1038/srep34036 27667442 PMC5036044

[42] Zhang L, Ren F, Zhang X, Wang X, Shi H, Zhou L, et al Peroxisome proliferator-activated receptor alpha acts as a mediator of endoplasmic reticulum stress-induced hepatocyte apoptosis in acute liver failure. Dis Model Mech. Company of Biologists; 2016;9: 799–809. 10.1242/dmm.023242 27482818 PMC4958306

[43] Cao A-L, Wang L, Chen X, Wang Y-M, Guo H-J, Chu S, et al Ursodeoxycholic acid and 4-phenylbutyrate prevent endoplasmic reticulum stress-induced podocyte apoptosis in diabetic nephropathy. Lab Investig. 2016;96: 610–622. 10.1038/labinvest.2016.44 26999661

[44] Hu H, Li L, Wang C, He H, Mao K, Ma X, et al 4-Phenylbutyric Acid Increases GLUT4 Gene Expression through Suppression of HDAC5 but not Endoplasmic Reticulum Stress. Cell Physiol Biochem. 2014;33: 1899–1910. 10.1159/000362967 25011668

[45] Wiley JC, Meabon JS, Frankowski H, Smith EA, Schecterson LC, Bothwell M, et al Phenylbutyric Acid Rescues Endoplasmic Reticulum Stress-Induced Suppression of APP Proteolysis and Prevents Apoptosis in Neuronal Cells. Combs C, editor. PLoS One. Public Library of Science; 2010;5: e9135 10.1371/journal.pone.0009135 20161760 PMC2817752

[46] Newmark HL, Young CW. Butyrate and phenylacetate as differentiating agents: Practical problems and opportunities. J Cell Biochem. Wiley Subscription Services, Inc., A Wiley Company; 1995;59: 247–253. 10.1002/jcb.240590831 8538206

